# Vascular endothelial dysfunction induced by 3-bromofluoranthene via MAPK-mediated-NFκB pro-inflammatory pathway and intracellular ROS generation

**DOI:** 10.1007/s00204-024-03751-0

**Published:** 2024-04-18

**Authors:** Chien-Ying Lee, Sheng-Wen Wu, Jiann-Jou Yang, Wen-Ying Chen, Chun-Jung Chen, Hsin-Hung Chen, Yi-Chia Lee, Chun-Hung Su, Yu-Hsiang Kuan

**Affiliations:** 1 Department of Pharmacology, School of Medicine, Chung Shan Medical University, No. 110, Sec. 1, Jianguo N. Rd., Taichung, 402 Taiwan, ROC; 2 Department of Pharmacy, Chung Shan Medical University Hospital, Taichung, Taiwan; 3 Division of Nephrology, Department of Internal Medicine, Chung Shan Medical University Hospital, Taichung, Taiwan; 4 Department of Internal Medicine, School of Medicine, Chung Shan Medical University, Taichung, Taiwan; 5 Department of BioMedical Sciences, Chung Shan Medical University, Taichung, Taiwan; 6 Department of Veterinary Medicine, National Chung Hsing University, Taichung, Taiwan; 7 Department of Education and Research, Taichung Veterans General Hospital, Taichung, Taiwan; 8 Division of Endocrinology and Metabolism, Department of Internal Medicine, Asia University Hospital, Taichung, Taiwan; 9 School of Medicine, Institute of Medicine and Public Health, Chung Shan Medical University, Taichung, Taiwan; 10 Chung Sheng Clinic, Nantou, Taiwan; 11 Department of Internal Medicine, Chung Shan Medical University Hospital, Taichung, Taiwan

**Keywords:** 3-Bromofluoranthene, Vascular endothelial dysfunction, MAPK, NFκB, ROS, Antioxidative enzyme, HO-1, Nrf-2

## Abstract

3-Bromofluoranthene (3-BrFlu) is the secondary metabolite of fluoranthene, which is classified as a polycyclic aromatic hydrocarbon, through bromination and exists in the fine particulate matter of air pollutants. Endothelial dysfunction plays a critical role in the pathogenesis of cardiovascular and vascular diseases. Little is known about the molecular mechanism of 3-BrFlu on endothelial dysfunction in vivo and in vitro assay. In the present study, 3-BrFlu included concentration-dependent changes in ectopic angiogenesis of the sub-intestinal vein and dilation of the dorsal aorta in zebrafish. Disruption of vascular endothelial integrity and up-regulation of vascular endothelial permeability were also induced by 3-BrFlu in a concentration-dependent manner through pro-inflammatory responses in vascular endothelial cells, namely, SVEC4-10 cells. Generation of pro-inflammatory mediator PGE2 was induced by 3-BrFlu through COX2 expression. Expression of COX2 and generation of pro-inflammatory cytokines, including TNFα and IL-6, were induced by 3-BrFlu through phosphorylation of NF-κB p65, which was mediated by phosphorylation of MAPK, including p38 MAPK, ERK and JNK. Furthermore, generation of intracellular ROS was induced by 3-BrFlu, which is associated with the down-regulated activities of the antioxidant enzyme (AOE), including SOD and catalase. We also found that 3-BrFlu up-regulated expression of the AOE and HO-1 induced by 3-BrFlu through Nrf-2 expression. However, the 3-BrFlu-induced upregulation of AOE and HO-1 expression could not be revised the responses of vascular endothelial dysfunction. In conclusion, 3-BrFlu is a hazardous substance that results in vascular endothelial dysfunction through the MAPK-mediated-NFκB pro-inflammatory pathway and intracellular ROS generation.

## Introduction

The high rate of morbidity and mortality for cardiovascular and vascular diseases appears to transcend that of cancers, respiratory bursts and other diseases, globally (Jagannathan et al. 2019; Ke et al. 2022). The normal function of the vascular endothelium is to promote the maintenance of the physiological state of the circulatory system, including the cardiovascular and vascular system. Pathological angiogenesis and upregulation of vascular permeability are important features of endothelial dysfunction (Sun et al. 2020). Vascular endothelial dysfunction is the cause of many diseases, including atherosclerosis, hypertension, coronary heart disease and diabetes (Wang et al. 2022a, b; Kong et al. 2022). Inflammation is the critical pathological feature of endothelial dysfunction diseases due to overproduction of oxidative stress and pro-inflammatory mediators such as prostaglandin (PG) E2 and pro-inflammatory cytokines, including interleukin (IL)-6 and tumor necrosis factor (TNF)-α (Chrissobolis et al. 2011; Sprague and Khalli 2009; Fu et al. 2017). Activation of nuclear factor (NF)-κB, the pro-inflammatory transcript factor, participates in the expression of pro-inflammatory responses via overexpression of reactive oxygen species (ROS) and phosphorylation of mitogen-activated protein kinase (MAPK), including extracellular signal-regulated kinase (ERK), p38 MAPK and c-Jun N-terminal kinase (JNK) (Zhenyukh et al. 2018; Rajendran et al. 2016). The intracellular antioxidative system, including superoxide dismutase (SOD), catalase and heme oxygenase (HO)-1, is triggered and detoxified by the harmful ROS (Hood et al. 2011).

Air pollution remains a serious health question to be the familiar with environmental health and climate threat. Fine particulate matter (PM2.5) is the major risk factor for cardiovascular disease due to vascular endothelial dysfunction (Liang et al. 2021; Hayes et al. 2020). Polycyclic aromatic hydrocarbons (PAHs) are primary organic and harmful compounds bound to PM2.5 that exist ubiquitously in indoor and outdoor environments (Pietrogrande et al. 2022; Hao et al. 2018). Fluoranthene is the predominant PAH in the combustion of final wastes during fossil fuel combustion, cooking and barbecuing and smoking (Abramsson-Zetterberg and Maurer 2015). 3-BrFlu is the secondary metabolite of fluoranthene via bromination that exists in air pollutants (Jin et al. 2017; Shi et al. 2020). Recent studies have shown that cardiotoxicity and vascular endothelial cell (VEC) apoptosis are achieved in zebrafish through the apoptotic pathway (Su et al. 2021). Therefore, the present study aims to explore whether 3-BrFlu affects vascular endothelial dysfunction and its underlying mechanisms, including the MAPK-mediated-NFκB pro-inflammatory pathway and the Nrf2-mediated AOE pathway.

## Methods

### Reagents and antibodies

Phosphate-buffered saline (PBS), fetal bovine serum (FBS), Dulbecco’s Modified Eagle Medium (DMEM), and antibiotic–antimycotic solution were purchased from Gibco (Grand Island, NY, USA). The antibodies used for the western bot analysis were as follows: cyclooxygenase-2 (COX-2), heme oxygenase-1 (HO-1), superoxide oxidase (SOD), catalase, β-actin, phosphoryl (P)-p65, p65, P-extracellular signal-regulated kinases (ERK), ERK, P–c-Jun N-terminal kinases (JNK), JNK, P-p38 MAPK, and p38 MAPK were purchased from Santa Cruz Biotechnology (Santa Cruz, CA, USA). Mouse IL-6, TNF-α, and PGE2 ELISA kits were purchased from R&D Systems (Minneapolis, MN, USA). Horseradish peroxidase‐conjugated secondary antibodies were obtained from Jackson ImmunoResearch Laboratories (Baltimore, MD, USA). Superoxide dismutase assay kit and catalase assay kit were obtained from were obtained from Cayman Chemical (Ann Arbor, MI, USA). 24-well Millicell Hanging Cell Culture Inserts with proe size of 8 μm and growth area of 0.33 cm^2^ were obtained from Merck Millipore (Burlington, MA, USA). Dimethyl sulfoxide (DMSO), 3-bromofluoranthene (3-BrFlu), bovine serum albumin (BSA), 2′,7′-dichlorofluorescein diacetate (DCFH-DA), and other reagents of analytical grade were supplied by Sigma-Aldrich (St. Louis, MO, USA). 3-BrFlu was dissolved in DMSO. The final concentration of DMSO was less than 0.5% (v/v) and had nontoxicity.

### Zebrafish maintenance

Transgenic (Tg[*fli-1: EGFP*]) zebrafish and embryos were collected from the Taiwan Zebrafish Core facility at Academia Sinica. The zebrafish were kept in recirculation tanks with a controlled light cycle of a 14 h light/10 h dark photoperiod at 28.5 °C and pH 7–8, as per the previous study (Su et al. 2021). The embryos were obtained through natural mating and collected in sea salt embryo media (including 5.03 mM sodium chloride, 0.17 mM potassium chloride, 0.003% 1-phenyl 2-thiourea, 0.33 mM calcium chloride, 0.33 mM magnesium sulfate, pH 7.4). All animal experiments were conducted in accordance with the Institutional Animal Care and Use Committee of Chung Shan Medical University (No. 2416).

### Pro-angiogenic effect and vascular changes of zebrafish

The selected 24-hpf zebrafish embryo were incubated with five concentrations of 3-BrFlu, including 0, 3, 10, 50 and 100 μM for 48 h. Each experimental condition contained at least 30 embryos per group. Zebrafish were observed and analysed under the Lionheart automated microscope (Biotek, Winooski, VT, USA). Three random points in the dorsal aorta (DA) of Tg*(fli1:EGFP)* zebrafish embryos were chosen for measurement of vessel diameter. The leading buds in the sub-intestinal vein (SIV) were chosen for measurement.

### Cell culture condition and treatment

SVEC4-10 endothelial cells were obtained from the Bioresource Collection and Research Center (Shinchu, Taiwan) and cultured in DMEM containing 10% FBS and 1% antibiotic–antimycotic solution in a humidified incubator maintained at 37 °C and 5% CO_2_. The culture medium was renewed after cell seeding or subculture for 48 h. After seeding, the SVEC4-10 cells were treated with 0, 3, 50 and 100 μM 3-BrFlu for 24 h in serum-free culture medium (Su et al. 2021).

### Transepithelial electrical resistance (TEER) measurement

SVEC4-10 cells were placed on the upper side of cell culture insert membranes with a pore size of 0.4 μm in the 24-well Transwell inserts. Following a 24 h incubation with varying concentrations of 3-BrFlu, the integrity of the endothelial monolayer in Transwells inserts was evaluated by measuring TEER with the Cica TEER measuring system (Kanto Chemical Co., Inc.) (Uzu and Takezawa 2020).

### Trans-endothelial albumin passage

SVEC4-10 cells were placed on the upper side of cell culture insert membranes with a pore size of 0.4 μm in the 24-well Transwell inserts. After incubating these cells with varying concentrations of 3-BrFlu for 24 h, 10% BSA was loaded on the upper side of the cell culture inserts and left for 6 h. The protein concentration in the bottom well was determined using the Bradford protein assay kit.

### PGE2, IL-6, TNFα, SOD activity and Catalase activity assays

After the SVEC4-10 cells were treated with 3-BrFlu, the contents of PGE2, IL-6 and TNFα in the culture medium were analysed using the IL-6, TNF-α and PGE2 ELISA kits, respectively. SOD and catalase were analysed using the superoxide dismutase assay kit and catalase assay kit, respectively. The procedures were carried out according to the manufacturer’s instructions (Yang et al. 2022a; b).

### Immunoblotting assay

To determine protein expression and phosphorylation, the immunoblotting assay was evaluated following a previously reported method (Chiang et al. 2022). After treatment of SVEC4-10 cells with 3-BrFlu, the total protein was extracted by RIPA lysis buffer with protease and phosphatase inhibitors. An equal amount of protein was separated by sodium dodecyl sulfate–polyacrylamide gel electrophoresis and electro-transferred onto polyvinylidene difluoride (PVDF) membranes. The PVDF membranes were blocked with 5% non-fat milk for 10 min on a shaking bed at room temperature. The PVDF membranes were reacted with primary antibodies against COX-2, HO-1, SOD, catalase, β-actin, P-p65, p65, P-ERK, ERK, P-JNK, JNK, P-p38 MAPK and p38 MAPK overnight at 4 °C. After washing, the PVDF membranes were incubated with horseradish peroxide-conjugated secondary antibody for 1 h at room temperature. After treatment with the electrochemiluminescence kit, the levels of protein expression and phorphorylation were measured using the Infiniti Vision System (Vilber, Lourmat, Collegien, France).

### Intracellular reactive oxygen species measurements

After treatment, SVEC4-10 cells were reacted with a DCFH-DA fluorescent probe at a concentration of 10 μM for 30 min at 37 °C. After washing with PBS, the DCF fluorescence intensity and level of ROS generation in the cells were measured using the Synergy HT multi-mode microplate reader (Biotek, Winooski, VT, USA) with excitation and emission wavelengths of 490 nm and 522 nm (Chiang et al. 2022).

### Statistical analysis

The results are shown as mean ± standard deviation of mean and were analysed using one-way analysis of variance (one-way ANOVA) followed by Bonferroni’s test for multiple comparisons. All analyses were performed using SPSS software (IBM, New York, NY, USA). *P* values less than 0.05 indicate a statistically significant difference.

## Results

### Effect of 3-BrFlu on ectopic angiogenesis and vasodilation in zebrafish embryos

**Fig. 1.**
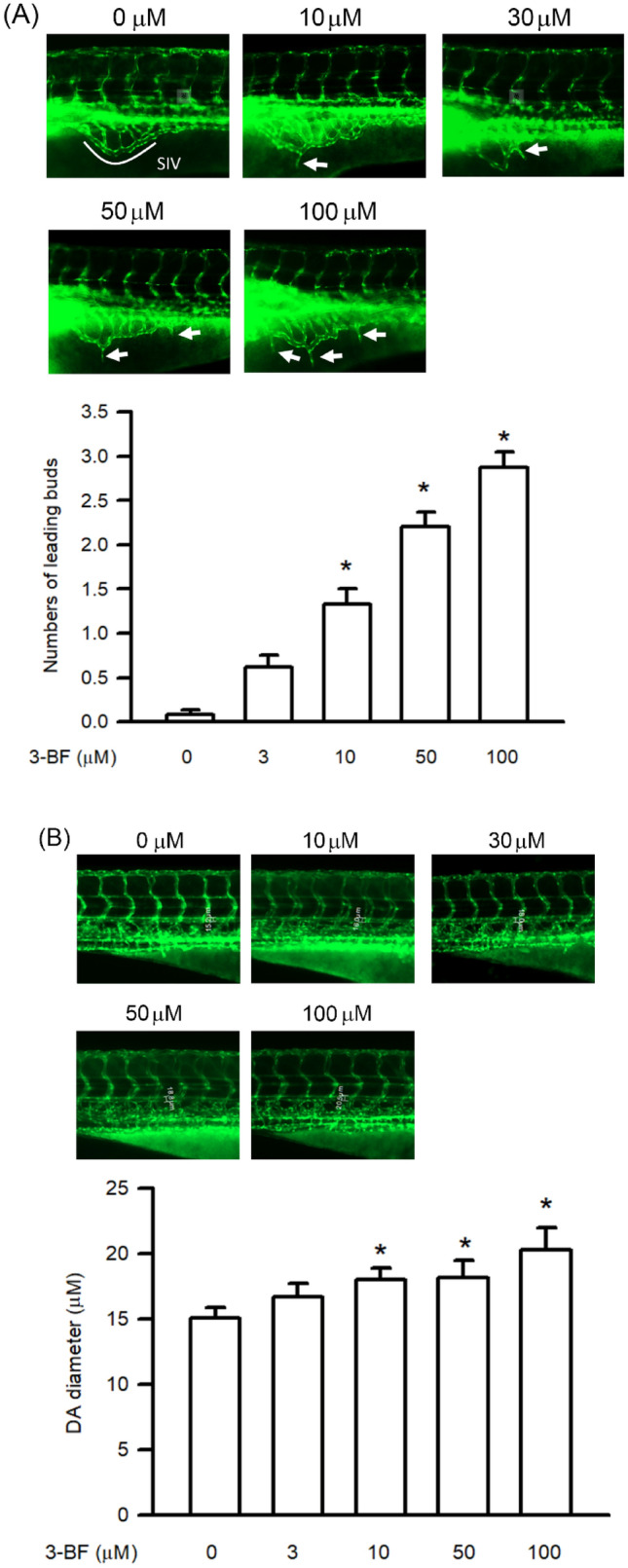
3-BrFlu induced ectopic angiogenesis and vasodilation in the Tg*(fli1:EGFP)* zebrafish embryo. The Tg*(fli1:EGFP)* zebrafish embryos were incubated with 3-BrFlu at concentration of 0, 3, 10, 50, and 100 μM from 24 to 72 hpf. **A** 3-BrFlu increased the number of ectopic angiogenesis in the SIV basket of zebrafish embryo. **B** 3-BrFlu increased the diameter of DA in zebrafish embryo. All data are represented as the mean ± standard deviation of the mean (*n* = 3). Significant differences (*P* < 0.05) between the various treatment groups are indicated by the differential letter at the top of the columns

Ectopic angiogenesis and relative vasodilation play a role as markers of endothelial dysfunction in the zebrafish model (Gibbs-Bar et al. 2016; Tang et al. 2010). In the Tg*(fli1:EGFP)* zebrafish embryo, the SIV was shown as a smooth basket-like structure after 0 μM 3-BrFlu treatment for 24 h. Following 3-BrFlu at 3, 10, 50 and 100 µM treatment from 24 to 72 hpf, ectopic angiogenesis formed bud-like projections from the SIV basket and increased in a concentration-dependent manner, with significant effects starting at 3 μM (*P* < 0.05, Fig. 1A). In addition, following 3-BrFlu at various concentration treatments, the diameter of DA increased in a concentration-dependent manner, with significant effects starting at 3 μM (*P* < 0.05, Fig. 1B).

### Effect of 3-BrFlu on vascular endothelial integrity and permeability in SVEC4-10 endothelial cells

**Fig. 2.**
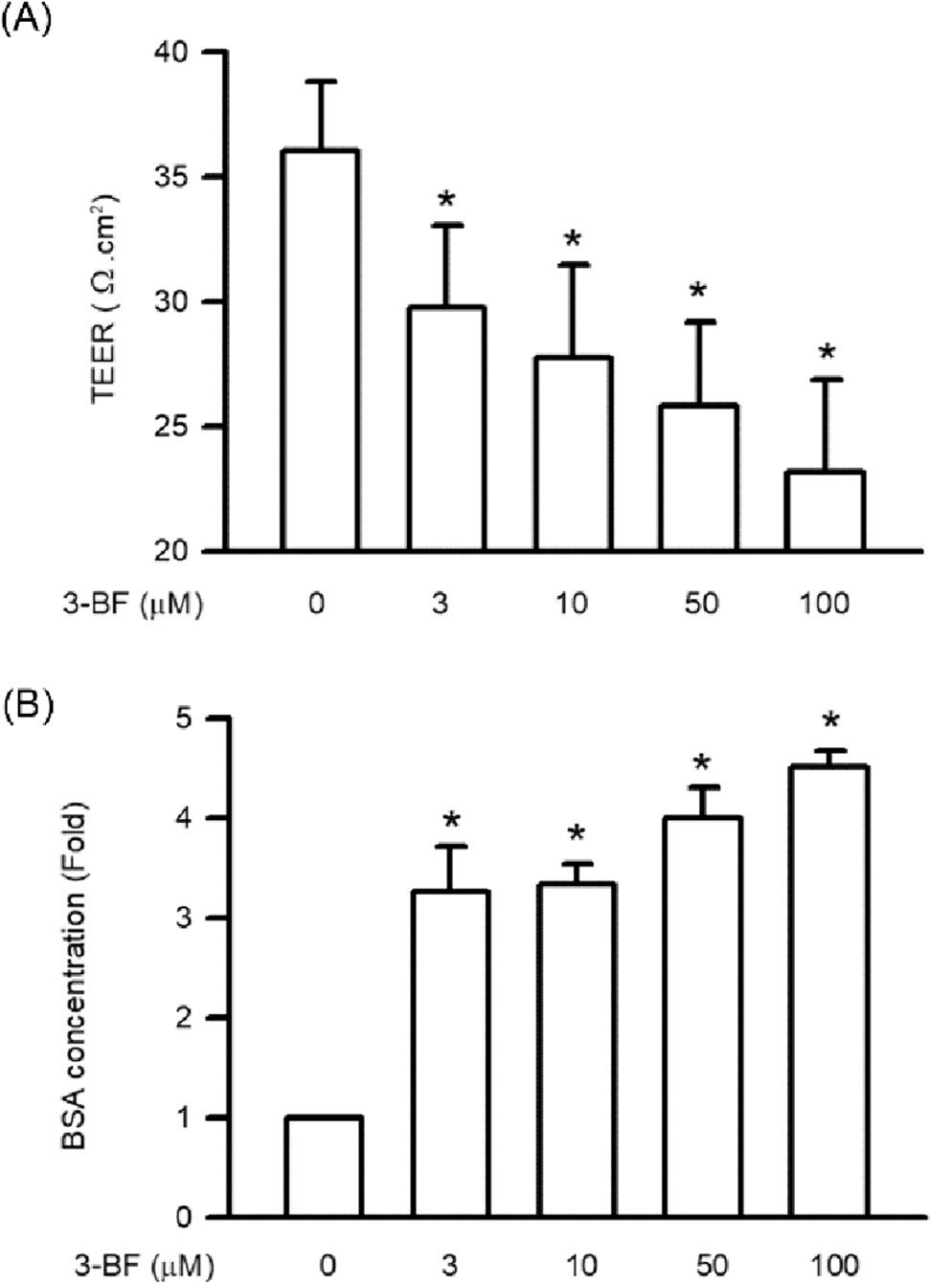
3-BrFlu induced integrity disruption and permeability increase in SVEC4-10 endothelial cells. The cells were incubated with 3-BrFlu at concentrations of 0, 3, 10, 50, and 100 μM for 24 h at 37 °C. **A** 3-BrFlu increased vascular endothelial integrity disruption through TEER measurements. **B** 3-BrFlu increased permeability through trans-endothelial albumin passage. All data are represented as the mean ± standard deviation of the mean (*n* = 3). Significant differences (*P* < 0.05) between the various treatment groups are indicated by the differential letter at the top of the columns

Downregulation of vascular endothelial integrity is the major step involved in ectopic angiogenesis and vasodilation due to upregulation of vascular endothelial permeability (LeGallo 2014). The effect of 3-BrFlu on vascular endothelial integrity and permeability in SVEC4-10 cells was assessed by TEER measurements and trans-endothelial albumin passage, respectively (Moll et al. 2021). As shown in Fig. 2A, 3-BrFlu altered vascular endothelial integrity in a concentration-dependent manner, with significant effects starting at 3 μM (*P* < 0.05). As shown in Fig. 2B, 3-BrFlu induced vascular endothelial permeability in a concentration-dependent manner, with significant effects starting at 3 μM (*P* < 0.05).

### Effects of 3-BrFlu on PGE2 generation and COX2 expression in SVEC4-10 endothelial cells

**Fig. 3.**
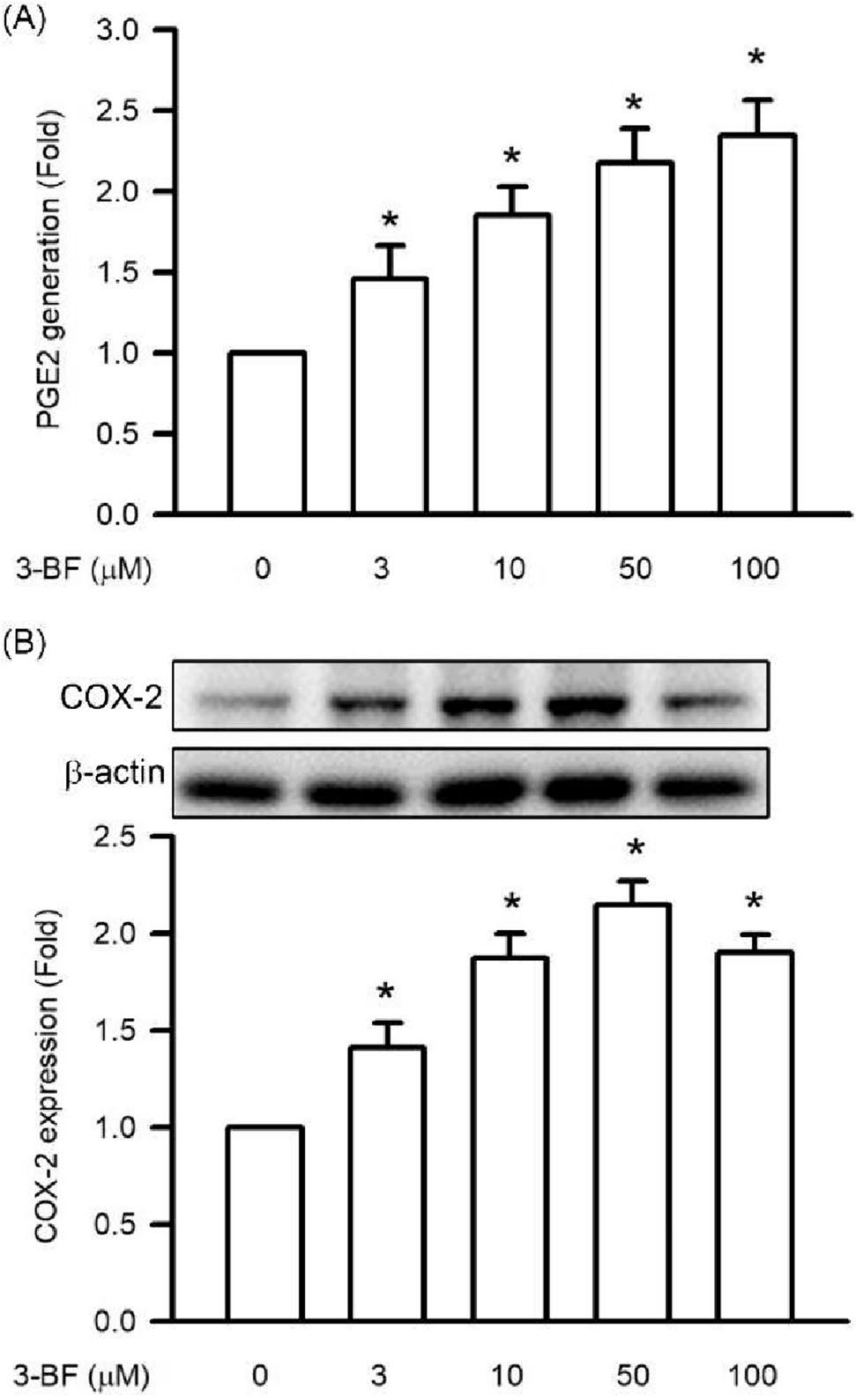
3-BrFlu-induced PGE2 generation and COX-2 expression in SVEC4-10 endothelial cells. The PGE2 generation and COX2 expression were measured by ELISA assay and immunoblotting assay after treated with 3-BrFlu for 24 h. The value of 0 μM 3-BrFlu treated SVEC4-10 cells for 24 h was set to 1. The exchange in fold of PGE2 generation and COX2 expression between the other treated and 0 μM 3-BrFlu-treated groups were calculated. All data are represented as the mean ± standard deviation of the mean (*n* = 3). Significant differences (*P* < 0.05) between the various treatment groups are indicated by the differential letter at the top of the columns

Generation of PGE2 and COX2 expression of SVEC4-10 endothelial cells incubated with 3-BrFlu at various concentrations was monitored for 24 h by ELISA and immunoblotting assay, respectively. 3-BrFlu-induced generation of PGE2 and COX2 expression occurred in a concentration-dependent manner, with significant effects observable from 3 μM (*P* < 0.05, Fig. 3). PGE2 generation and COX2 expression were significantly increased in fold from 1.00 (control) to 1.46–2.35 and 1.41–1.91, respectively, by 3–100 μM 3-BrFlu. These results indicate that 3-BrFlu induced PGE2 generation and COX2 expression in SVEC4-10 endothelial cells.

### Effects of 3-BrFlu on expression of IL-6 and TNFα in SVEC4-10 endothelial cells

**Fig. 4.**
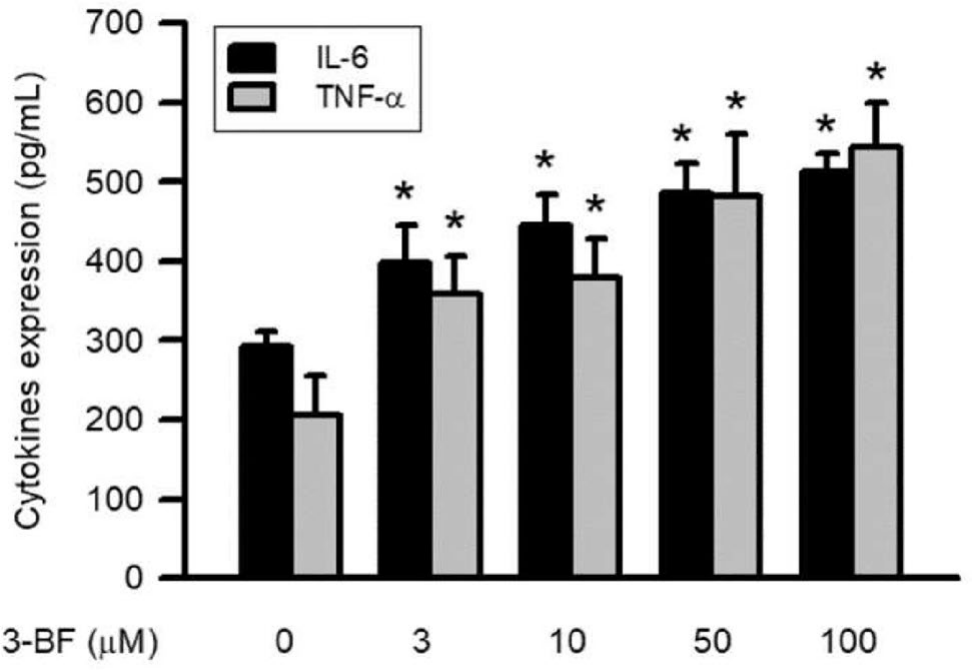
3-BrFlu-induced generation of IL-6 and TNFα in SVEC4-10 endothelial cells. Generation of IL-6 and TNFα were measured by ELISA assay after treated with 3-BrFlu for 24 h. The value of 0 μM 3-BrFlu treated SVEC4-10 cells for 24 h was set to 1. The exchange in fold of IL-6 and TNFα generation between the other treated and 0 μM 3-BrFlu-treated groups were calculated. All data are represented as the mean ± standard deviation of the mean (*n* = 3). Significant differences (*P* < 0.05) between the various treatment groups are indicated by the differential letter at the top of the columns

Cytokine expression, including of IL-6 and TNFα, of SVEC4-10 endothelial cells incubated with 3-BrFlu at various concentrations was monitored for 24 h by immunoblotting assay. 3-BrFlu-induced IL-6 and TNFα expression occurred in a concentration-dependent manner, with significant effects observable beginning at 3 μM (*P* < 0.05, Fig. 4). IL-6 expression was significantly increased from 293.53 (pg/mL) to 398.56–513.75 and 206.74 (pg/mL) by 3–100 μM 3-BrFlu. TNFα expression was significantly increased from 206.74 (pg/mL) to 3.58.42–543.21 (pg/mL) by 3–100 μM 3-BrFlu. These results indicate that IL-6 and TNFα expression was induced by 3-BrFlu in SVEC4-10 endothelial cells.

### Effects of 3-BrFlu on NFκB p65 phosphorylation in SVEC4-10 endothelial cells

**Fig. 5.**
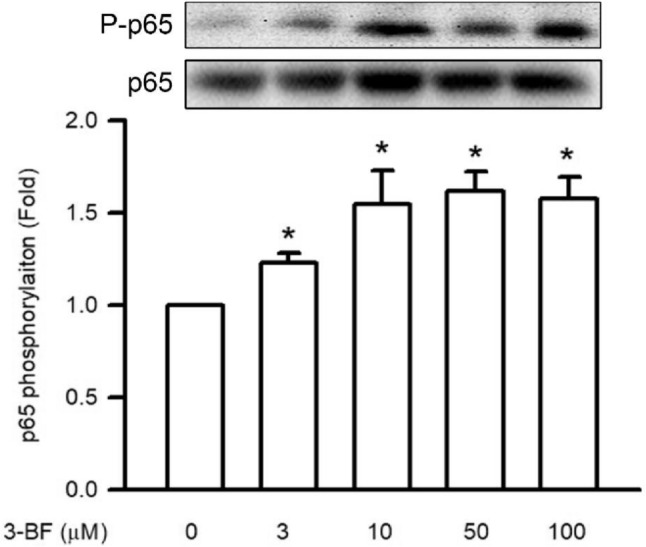
3-BrFlu-induced NFκB p65 phosphorylation in SVEC4-10 endothelial cells. Phosphorylation of NFκB p65 was measured by immunoblotting assay after treated with 3-BrFlu for 24 h. The value of 0 μM 3-BrFlu treated SVEC4-10 cells for 24 h was set to 1. The exchange in fold of NFκB p65 phosphorylation between the other treated and 0 μM 3-BrFlu-treated groups were calculated. All data are represented as the mean ± standard deviation of the mean (*n* = 3). Significant differences (*P* < 0.05) between the various treatment groups are indicated by the differential letter at the top of the columns

Expression of pro-inflammatory mediator is regulated by the phosphorylation of NFκB p65, which was monitored through the immunoblotting assay. 3-BrFlu-induced NFκB p65 phosphorylation occurred in a concentration-dependent manner, with significant effects observable beginning at 3 μM (*P* < 0.05, Fig. 5). NFκB p65 phosphorylation was significantly increased in fold from 1.00 to 1.23–1.58 by 3–100 μM 3-BrFlu. These results indicate that NFκB p65 phosphorylation was induced by 3-BrFlu in SVEC4-10 endothelial cells.

### Effects of 3-BrFlu on MAPK phosphorylation in SVEC4-10 endothelial cells

**Fig. 6.**
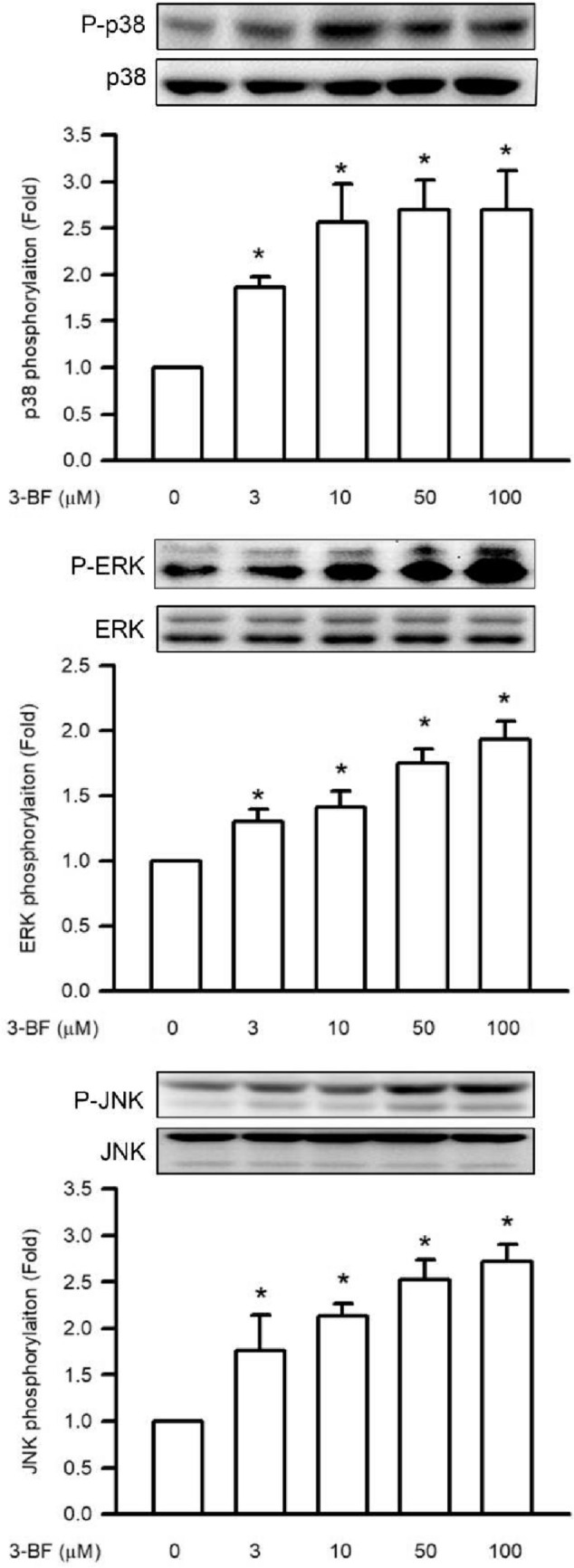
3-BrFlu-induced MAPK phosphorylation in SVEC4-10 endothelial cells. Phosphorylation of p38 MAPK, ERK, JNK was measured by immunoblotting assay after treated with 3-BrFlu for 24 h. The value of 0 μM 3-BrFlu treated SVEC4-10 cells for 24 h was set to 1. The exchange in fold of MAPK phosphorylation between the other treated and 0 μM 3-BrFlu-treated groups were calculated. All data are represented as the mean ± standard deviation of the mean (*n* = 3). Significant differences (*P* < 0.05) between the various treatment groups are indicated by the differential letter at the top of the columns

Phosphorylation of MAPK, including p38 MAPK, ERK and JNK, is the important upstream factor of NFκB p65. Phosphorylation of MAPK was monitored through the immunoblotting assay. 3-BrFlu-induced MAPK phosphorylation occurred in a concentration-dependent manner, with significant effects observed beginning at 3 μM (*P* < 0.05, Fig. 6). p38 MAPK, ERK and JNK phosphorylation was significantly increased in fold from 1.86 to 2.69, 1.31–1.94 and 1.77–2.73, respectively, by 3–100 μM 3-BrFlu. These results indicate that MAPK phosphorylation was induced by 3-BrFlu in SVEC4-10 endothelial cells.

### Effects of 3-BrFlu on intracellular ROS accumulation in SVEC4-10 endothelial cells

**Fig. 7.**
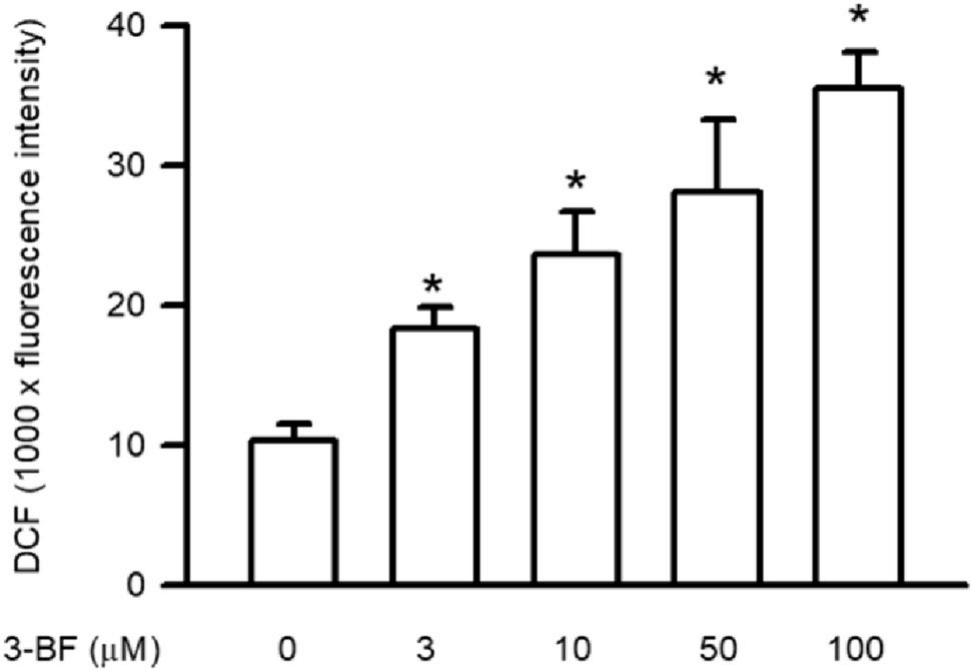
3-BrFlu-induced intracellular ROS accumulation in SVEC4-10 endothelial cells. Intracellular ROS accumulation was measured by DCFH-DA assay after treated with 3-BrFlu for 24 h. The value of 0 μM 3-BrFlu treated SVEC4-10 cells for 24 h was set to 1. The fluorescence intensity of each group was represented as mean ± standard deviation of the mean (*n* = 3). Significant differences (*P* < 0.05) between the various treatment groups are indicated by the differential letter at the top of the columns

Intracellular ROS accumulation has a critical role in vascular barrier function. Intracellular ROS accumulation was monitored through a DCFH-DA fluorescent probe. Accumulation of intracellular ROS occurred in a concentration-dependent manner, with significant effects observable beginning at 3 μM (*P* < 0.05, Fig. 7). Intracellular ROS accumulation was significantly increased in fluorescence intensity from 1.04 × 10^4^ to 1.83 × 10^4^–3.56 × 10^4^ by 10–100 μM 3-BrFlu. These results indicate that intracellular ROS accumulation was induced by 3-BrFlu in SVEC4-10 endothelial cells.

### Effects of 3-BrFlu on activities of SOD and catalase in SVEC4-10 endothelial cells

**Fig. 8.**
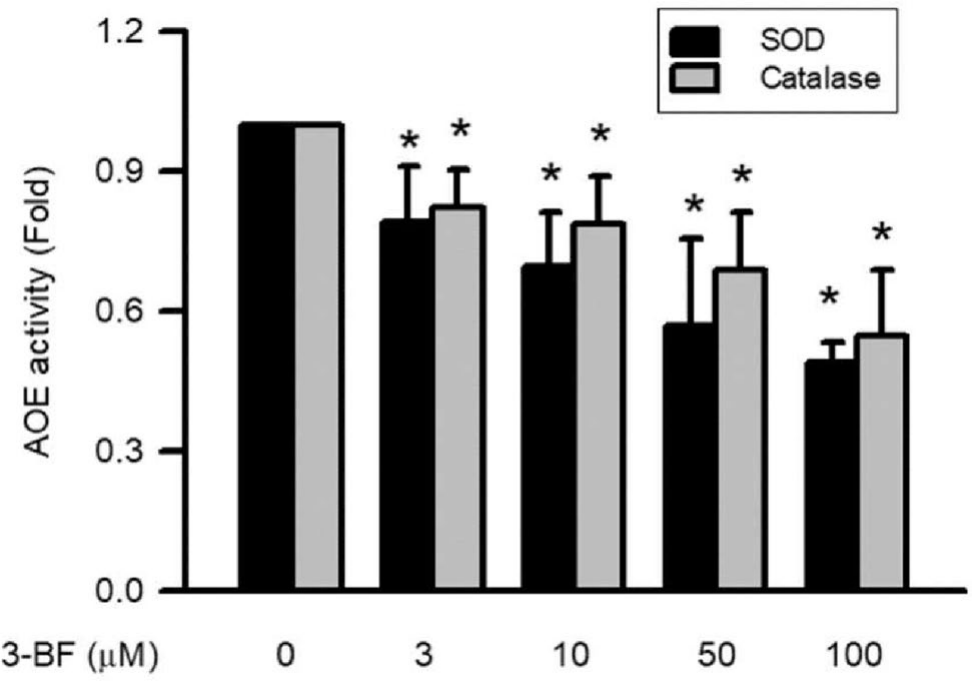
3-BrFlu-induced activities of SOD and catalase in SVEC4-10 endothelial cells. The value of 0 μM 3-BrFlu treated SVEC4-10 cells for 24 h was set to 1. The exchange in fold of SOD and catalase activities between the other treated and 0 μM 3-BrFlu-treated groups were calculated. All data are represented as the mean ± standard deviation of the mean (*n* = 3). Significant differences (*P* < 0.05) between the various treatment groups are indicated by the differential letter at the top of the columns

The 3-BrFlu-reduced the activities of SOD and catalase occurred in a concentration-dependent manner, with significant effects observable beginning at 3 μM (*P* < 0.05, Fig. 8). The activities of SOD and catalase was significantly decreased in fold from 1.00 to 0.79–0.48 and 0.82–0.55, respectively, by 3–100 μM 3-BrFlu. These results indicated that the activities of SOD and catalase were decreased by 3-BrFlu in SVEC4-10 endothelial cells.

### Effects of 3-BrFlu on expression of SOD and catalase in SVEC4-10 endothelial cells

**Fig. 9.**
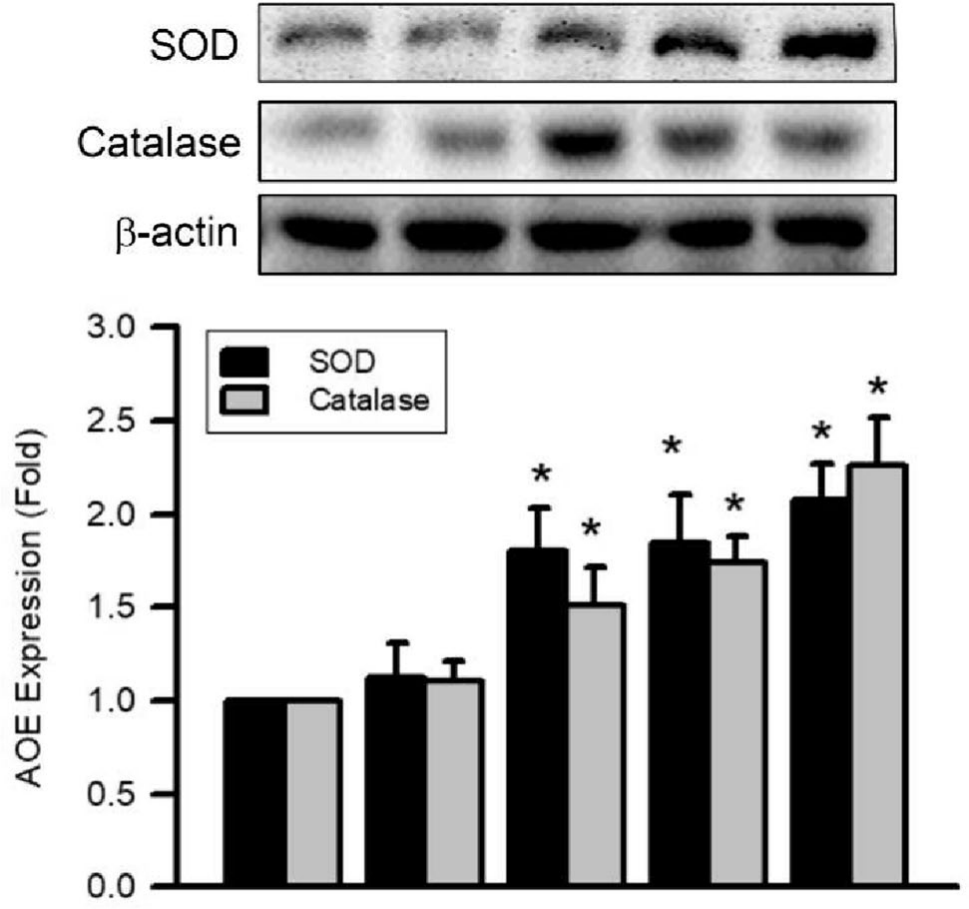
3-BrFlu-induced SOD and catalase expression in SVEC4-10 endothelial cells. Expression of SOD and catalase was measured by immunoblotting assay after treated with 3-BrFlu for 24 h. The value of 0 μM 3-BrFlu treated SVEC4-10 cells for 24 h was set to 1. The exchange in fold of SOD and catalase expression between the other treated and 0 μM 3-BrFlu-treated groups were calculated. All data are represented as the mean ± standard deviation of the mean (*n* = 3). Significant differences (*P* < 0.05) between the various treatment groups are indicated by the differential letter at the top of the columns

Expression of SOD and catalase was monitored through immunoblotting assay. 3-BrFlu-induced expression of SOD and catalase occurred in a concentration-dependent manner, with significant effects observable beginning at 10 μM (*P* < 0.05, Fig. 9). Expression of SOD and catalase was significantly increased in fold, from 1.00 to 1.80–2.07 and 1.51–2.26, respectively, by 10–100 μM 3-BrFlu. These results indicate that expression of SOD and catalase was induced by 3-BrFlu in SVEC4-10 endothelial cells.

### Effects of 3-BrFlu on expression of HO-1 and Nrf-2 in SVEC4-10 endothelial cells

**Fig. 10.**
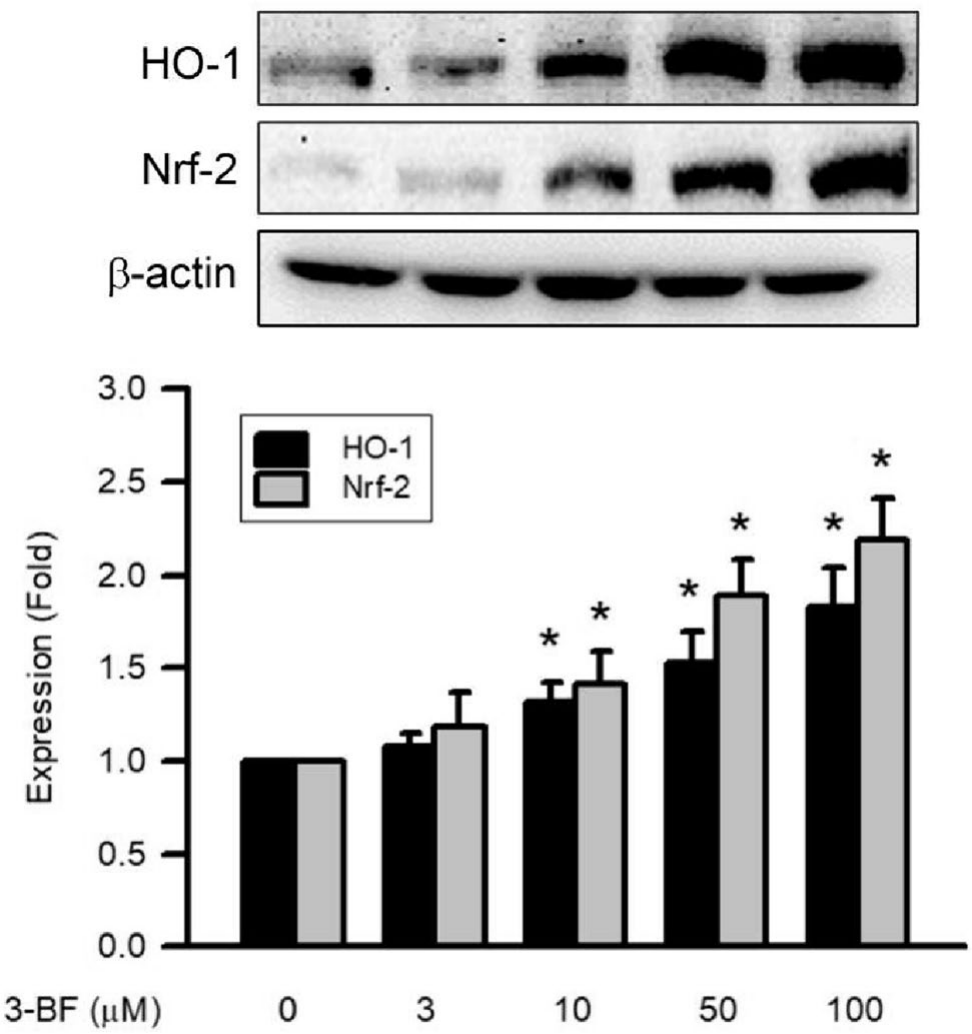
3-BrFlu-induced HO-1 and Nrf-2 expression in SVEC4-10 endothelial cells. Expression of HO-1 and Nrf-2 was measured by immunoblotting assay after treated with 3-BrFlu for 24 h. The value of 0 μM 3-BrFlu treated SVEC4-10 cells for 24 h was set to 1. The exchange in fold of HO-1 and Nrf-2 expression between the other treated and 0 μM 3-BrFlu-treated groups were calculated. All data are represented as the mean ± standard deviation of the mean (*n* = 3). Significant differences (*P* < 0.05) between the various treatment groups are indicated by the differential letter at the top of the columns

The 3-BrFlu-induced expression of HO-1 and Nrf-2 occurred in a concentration-dependent manner, with significant effects observable beginning at 1 10 μM (*P* < 0.05, Fig. 10). Expression of HO-1 and Nrf-2 increased significantly from 1.00 to 1.32–1.83 and 1.42–2.19, respectively, by 10–100 μM 3-BrFlu. These results indicate that expression of HO-1 and Nrf-2 was induced by 3-BrFlu in SVEC4-10 endothelial cells.

## Discussion

3-BrFlu is the halogenated component of the fluoranthene content in the environment and has been identified as a marker for food cooking, wood burning, incineration and oil combustion (Jin et al. 2017; Shi et al. 2020; Abramsson-Zetterberg and Maurer 2015). The pro-inflammatory response, oxidative stress and DNA damage were up-regulated after PM2.5 that contained fluoranthene in mice (de Oliveira Alves et al. 2020). In BALB/c mice, PAH induced endothelial cells, including the expression of adhesion molecules such as ICAM-1, VCAM-1 and E-selectin, in aortic tissue, and the generation of pro-inflammatory cytokines, including IL-6, TNF-α and INF-γ, in serum were induced by PAH composed of 55% phenanthrene, 25% fluoranthene and 20% pyrene (Rojas et al. 2022). Furthermore, we also found that 3-BrFlu induces apoptosis through the caspase-dependent pathway in endothelial cells, SVEC4-10 cells and cardiotoxicity in zebrafish (Su et al. 2021). These findings led us to explore further the 3-BrFlu-induced endothelial dysfunction in zebrafish and the molecular mechanism in SVEC4-10 cells. Ectopic angiogenesis is the important marker for vascular endothelial dysfunction and cardiovascular disease risk factor in the zebrafish model (Gibbs-Bar et al. 2016). Vasodilation is the main precursor of ectopic angiogenesis in zebrafish (Tang et al. 2010). SVEC4-10 cells are an immortalized cell line of murine endothelial cells that were isolated from the vascular epithelium of an adult male mouse. They have been widely used in research on endothelial cell dysfunction and proinflammation (Su et al. 2021). We primarily found that ectopic angiogenesis of SIV and dilation of DA in zebrafish after 3-BrFlu treatment. These results indicate that 3-BrFlu could have a potential effect in vascular endothelial dysfunction in the zebrafish model.

Under physiological conditions, the vascular barrier is sealed by paracellular endothelial cells via the tight junction (Radeva and Waschke 2018). Vascular endothelial dysfunction has been implicated in plaque progression, atherogenesis, stroke, coronary heart disease and hypertension (Wang et al. 2022a, b; Kong et al. 2022; Lawal et al. 2016). Upregulation of permeability in VEC is the initial and critical step in vascular endothelial dysfunction that involves vasodilation and then leads to ectopic angiogenesis (LeGallo 2014; Lawal et al. 2016). The vascular endothelial permeability is increased by exposure to PM2.5, which contains PAH and fluoranthene, in vivo and in vitro assay (Dai et al. 2017). We began by finding disruption of vascular endothelial integrity and upregulation of vascular endothelial permeability in SVEC4-10 cells treated with 3-BrFlu in a concentration-dependent manner. These results indicate that 3-BrFlu has the effect of vascular endothelial dysfunction caused by the disruption of integrity and upregulation permeability of endothelial cell permeability.

Inflammation plays an important role in defence against foreign pathogens in the human body (Marshall et al. 2018). Vascular endothelial dysfunction is an abnormal inflammatory process that includes the overproduction of pro-inflammatory mediators such as PGE2 and cytokines, including IL-6 and TNF-α (Sun et al. 2020; Yin et al. 2018; Tudorache et al. 2022). PGE2 induces the initial role in the inflammatory response to vascular hyperpermeability through activation of the EP2 and EP4 receptors (Omori et al. 2014). Furthermore, pro-inflammatory agonists induce PGE2 secretion through the expression of COX-2, which is the inducible enzyme (Omori et al. 2014; Yin et al. 2017). PM2.5-induced COX-2 expression and PGE2 secretion result in dysfunction and harmful response in VEC (Yin et al. 2017). Furthermore, COX-2 expression and PGE2 secretion are induced by fluoranthene at a concentration of 62.5 μM in human keratinocytes and HaCaT cells (Alalaiwe et al. 2020). In contrast to our research, 3-BrFlu at a concentration of 3 μM induced COX-2 expression and PGE2 secretion in SVEC-10 cells. Based on these results, we could suggest that the capacity of COX-2 expression induced by 3-BrFlu is higher than fluoranthene. Upon detection of damage- or pathogen-associated molecular patterns, IL-6 and TNFα are the major pro-inflammatory cytokines involved in vascular endothelial dysfunction (Kang and Kishimoto 2021; Sawant et al. 2013). After a PAH mixture that contains fluoranthene exposure, the contents of TNFα and IL-6 are upregulated in human umbilical vein endothelial cells (HUVEC) and the serum of BALB/c mice (Rojas et al. 2022; He et al. 2022). After exposure to 3-BrFlu for 24 h, the secretion of TNFα and IL-6 was significantly increased in a concentration-dependent manner in SVEC4-10 cells. Based on these findings, we propose that 3-BrFlu induces vascular endothelial dysfunction in zebrafish and endothelial cells through the overproduction of pro-inflammatory mediators and cytokines.

The stress-activated transcription factor NF-κB has been shown to play a functional role in inflammatory processes resulting in vascular endothelial dysfunction (Fan et al. 2022). Phosphorylation of MAPK, including p38 MAPK, ERK and JNK, is the important upstream protein molecule of NF-κB (Rajendran et al. 2016). Phosphorylation of NFκB p65 and MAPK is fluoranthene-containing PAH mixture and PM2.5 in HUVEC and visceral adipose tissue (He et al. 2022; Pan et al. 2019; Rui et al. 2016). Phosphorylation of p38 MAPK, ERK and JNK is induced by fluoranthene at a concentration of 45 μM after treatment for 0.5 h in the mouse non-tumorigenic alveolar type II cell line and C10 cells (Osgood et al. 2017). Our work demonstrates that phosphorylation of NFκB p65, p38 MAPK, ERK and JNK is significantly induced by 3-BrFlu at a concentration of 3 μM for 24 h in SVEC4-10 cells. Thus, we suggest that the ability of MAPK phosphorylation induced by 3-BrFlu is higher than that of fluoranthene. The present results indicate that the 3-BrFlu-induced pro-inflammatory response is mediated by the phosphorylation of NFκB p65 through the phosphorylation of MAPK in SVEC4-10 cells.

Oxidative stress, caused by the accumulation of intracellular ROS, can lead to various diseases such as atherosclerosis, diabetes mellitus, Alzheimer's disease, and Parkinson's disease due to VEC proinflammation and dysfunction. (Li et al. 2023; Ravi et al. 2024; Wang et al. 2023). Intracellular ROS production contributes to the pro-inflammatory and dysfunctional state of VECs through the activation of NF-κB (Zhenyukh et al. 2018). Antioxidative system includes SOD, catalase, and HO-1 to neutralize and detoxify intracellular ROS overproduction (Hood et al. 2011). SOD converts superoxide radicals to molecular oxygen and hydrogen peroxide, while catalase catalyses hydrogen peroxide into oxygen and water. HO-1, the ROS inducible enzyme, is the rate-limiting enzyme in the degradation of heme to carbon monoxide, iron, biliverdin and bilirubin (Thomas et al. 2022). In previous studies, intracellular ROS generation was induced by PM2.5 containing fluoranthene and sourced from cooking oil fumes or urban environments and a PAH mixture containing fluoranthene in HUVEC (He et al. 2022). Additionally, the downregulation of SOD activity and the upregulation of HO-1 expression were obtained in HUVEC after exposure to fluoranthene-containing PAH mixture (He et al. 2022). Here, our data also showed that intracellular ROS is generated after exposure to 3-BrFlu in a concentration-dependent manner in SVEC4-10 cells, and the activities of antioxidative enzymes (AOE), SOD and catalase are decreased by 3-BrFlu in a concentration-dependent manner. Furthermore, the expression of the antioxidative system, including SOD, catalase and HO-1, is dependent on the concentration. These observations support the hypothesis that vascular endothelial inflammation through intracellular ROS generation associated with down-regulation of AOE activities and could not revised by up-regulation of the AOE and HO-1 expression.

The expression of the antioxidant system is regulated by the activation of Nrf-2, which is the transcription factor activated by oxidative stress, in endothelial cells (Alonso-Piñeiro et al. 2021). In the resting state, the unactivated Nrf-2 binds to Kelch-like ECH-associated protein 1 (Keap1) to assemble a stable Nrf-2/Keap1 complex that existed in the cytoplasm. In a situation of oxidative stress stimulation, Nrf-2 is dissociated from the Nrf-2/Keap1 complex due to phosphorylation. The translocation of Nrf-2 phosphorylated into nuclei and binding to target genes for the antioxidant response element target genes lead to the expression of the antioxidative system that includes SOD, catalase and HO-1. After stimulation of oxidative stress, cytoplasmic or unactivated Nrf-2 is degraded through the ubiquitin–proteasome pathway (Alonso-Piñeiro et al. 2021). Nrf-2 expression is induced by the fluoranthene-containing PAH mixture in HUVEC (He et al. 2022). In the present study, 3-BrFlu induced Nrf-2 expression in a concentration-dependent manner in SVEC4-10 cells. According to the present results, 3-BrFlu induces the expression of the antioxidative system through the expression of Nrf-2.

This study has some limitations. Primarily, the use of the zebrafish animal model and the SVEC4-10 cell model to assess 3-BrFlu effects may not directly translate to human vascular responses due to species-specific differences. Additional research using human-based models is necessary to confirm the relevance of our findings to human health. Furthermore, our investigation focused on specific molecular pathways, including the MAPK-mediated-NFκB pathway, ROS generation, and caspase-dependent apoptosis, to elucidate the cardiovascular impacts of 3-BrFlu. This approach does not encompass all possible mechanisms contributing to cardiovascular dysfunction. The complexity of cardiovascular disease suggests other significant pathways may be involved. Further studies are needed to explore these avenues and fully understand the biological effects of 3-BrFlu. Overall, while our results provide insights into the cardiovascular implications of 3-BrFlu, they should be considered preliminary. Future research should aim to validate these findings in human contexts and uncover additional implicated molecular mechanisms.

## Conclusion

**Fig. 11:**
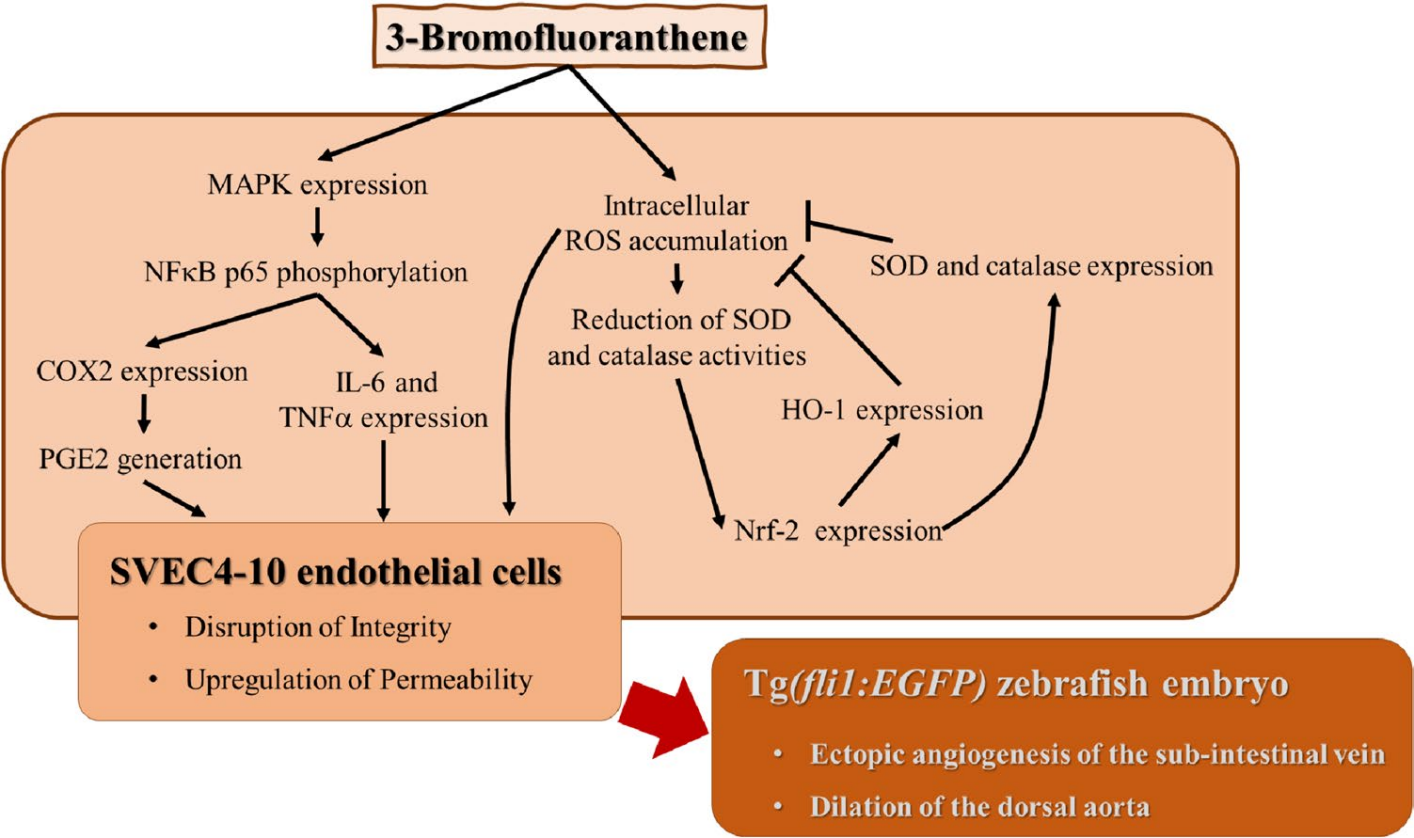
Schemes of the mechanism for vascular endothelial dysfunction induced by 3-bromofluoranthene in the zebrafish and SVEC4-10 endothelial cell

We began by proposing that 3-BrFlu induces endothelial dysfunction in the zebrafish model and the vascular endothelial cells (SVEC4-10) model, as shown in Fig. 11. Ectopic angiogenesis of SIV and dilation of DA were induced by 3-BrFlu in zebrafish. Disruption of vascular endothelial integrity and upregulation of vascular endothelial permeability were induced by 3-BrFlu in SVEC4-10 cells. Endothelial dysfunction induced by 3-BrFlu was mediated by upregulation of pro-inflammatory responses, such as an increase in PGE2 generation via upregulation of COX2 expression, promotion of pro-inflammatory cytokines TNFα and IL-6 generation and overproduction of intracellular ROS generation. The molecular mechanism of 3-BrFlu-induced pro-inflammatory responses is regulated by phosphorylation of NF-κB and its upstream factor p38 MAPK, ERK and JNK phosphorylation. On the other hand, overproduction of intracellular ROS generation induced by 3-BrFlu is associated with downregulation of AOE, SOD and catalase activities. Although upregulated expression of the AOE and HO-1 is induced by 3-BrFlu via Nrf-2 expression, the responses of vascular endothelial dysfunction could not be resolved by the AOE and HO-1. Our results suggest that 3-BrFlu is a pro-inflammatory response induced by hazardous substance-induced pro-inflammatory response via MAPK-mediated-NFκB pathway and intracellular ROS in vascular endothelial cells that results in the endothelial dysfunction in the zebrafish model and the cellular model.

## Data Availability

The data that support the findings of this study are available from the corresponding author, Yu-Hsiang Kuan, upon reasonable request.
